# Sex-Specific Seasonal Trajectories of Photosystem II Function During Natural Senescence in *Ginkgo biloba* Revealed by OJIP Fluorescence Analysis

**DOI:** 10.3390/life16071060

**Published:** 2026-06-25

**Authors:** Fanghao Cheng, Mei He, Xinyuan Lao, Kaimei Zhang, Dawei Shi

**Affiliations:** 1 College of Life Sciences, Nanjing Forestry University, Nanjing 210037, China; cfh2002@njfu.edu.cn (F.C.); 2311801209@njfu.edu.cn (X.L.); 2 Jiangxi Academy of Forestry, Nanchang 330032, China; sangelgirp1365@126.com

**Keywords:** dioecy, *Ginkgo biloba*, natural leaf senescence, photosystem II, photosynthetic performance, OJIP fluorescence, JIP-test

## Abstract

Dioecious plants often exhibit sex-specific physiological strategies that influence their response to environmental change. However, it is not well understood whether such dimorphism extends to the developmental trajectory of the photosynthetic apparatus during natural senescence. In this study, we compared the seasonal development and decline of photosystem II (PSII) function in naturally grown male and female *Ginkgo biloba* using non-destructive fast chlorophyll a fluorescence induction kinetics (OJIP) and JIP-test analysis. Sun-exposed, healthy leaves were sampled at approximately 15-day intervals from 18 July to 26 November 2024 [day of year, (DOY 188–332)]. The study monitored chlorophyll content and OJIP-derived parameters, and evaluated sex differences statistically (*p* < 0.05). Chlorophyll content began to decline after DOY 268 in both sexes, but decreased earlier and more rapidly in males. By DOY 332, male chlorophyll content fell to 1.37% of its level at DOY 268, whereas females retained 9.55%. OJIP fluorescence transient analysis revealed that ΔW_oj_ shifted from negative to positive values after DOY 268 in male plants, accompanied by a sustained increase in the relative variable fluorescence at the J step (V_j_). This pattern indicates an earlier and more pronounced acceptor-side limitation of PSII in male plants, associated with accelerated accumulation of QA^−^ and restricted electron transfer from QA^−^ to QB and the plastoquinone (PQ) pool. In addition, male plants showed a clearer donor-side limitation, with a pronounced ΔW_ok_ response, suggesting reduced stability of the oxygen-evolving complex (OEC). In contrast, females maintained higher cross-section-based energy fluxes (TR_0_/CS_0_, ET_0_/CS_0_) and PSI-end acceptor reduction capacity (RE_0_/CS_0_), and exhibited a slower decline in integrated performance indices (PI abs, PI total, DF abs). Principal component analysis further suggested that male senescence trajectories were more tightly associated with changes in electron-transport efficiency, whereas females exhibited a more gradual adjustment in energy-flux allocation. Collectively, these results reveal pronounced sexual dimorphism in the PSII–PSI functional decline pathway during natural senescence in *G. biloba* and provide a physiological basis for understanding sex-specific variation in photosynthetic decline in this species, with potential relevance to broader studies of dioecious plants.

## 1. Introduction

Dioecious plants represent a distinctive group within the plant kingdom. They play important roles in maintaining ecological stability and biodiversity. Over the course of long-term growth and evolution, dioecious plants have developed pronounced sexual differences in their responses to environmental stresses, with distinct morphological traits and physiological regulatory strategies often observed between male and female individuals. For example, under conditions of imbalanced nitrogen (N) and phosphorus (P) deposition, male and female *Populus cathayana* exhibit significant sexual dimorphism in the accumulation of chemical compounds [1]. Therefore, exploring sexual dimorphism in dioecious plants not only deepens our understanding of the mechanisms underlying plant growth, reproduction, and environmental adaptation, but also provides an important theoretical basis for plant breeding, ecological restoration, and biodiversity conservation.

Plant growth and development depend on photosynthesis. Photosynthesis is the material basis of all material and energy metabolism in the biosphere. It involves a series of complex photophysical, photochemical and biochemical reactions. Photosystem II (PSII) is considered the most vulnerable component of the photosynthetic apparatus. During plant senescence, the photosynthetic rate usually undergoes significant changes [2]. Chlorophyll fluorescence measurement provides a non-destructive method for detecting the photochemical parameters of PSII. This technique offers an effective approach for studying the functional status of the photosynthetic electron transport chain and the performance of PSII during leaf development, and it also provides an important basis for evaluating plant growth. At present, chlorophyll fluorescence analysis has been widely used to assess the degree of plant damage caused by biotic and abiotic stresses in many species, including *Oryza sativa* [3], *Populus euphratica* [4], and *Valeriana jatamansi* [5]. In addition, it has also been applied in studies of natural plant senescence [6,7,8].

*Ginkgo biloba* is one of the oldest tree species on Earth and is often referred to as a “living fossil” in the plant kingdom. Among extant gymnosperms, *Ginkgo biloba*, as an ancient relict species, retains many primitive characteristics. Some of these features are relatively rare in gymnosperms but are common in ferns [9]. In addition, as a dioecious plant, male and female individuals of *Ginkgo biloba* exhibit significant differences in many aspects. For instance, Li et al. [10] documented significant differences between male and female trees in diameter at breast height (DBH), clear bole height (CBH), tree height, and stem volume.

Recent studies have documented sex-specific differences in photosynthetic performance and physiological traits of *Ginkgo* under both stress and natural conditions. Under salt stress, chlorophyll fluorescence parameters (e.g., F_v_/F_m_ and ΦPSII) as well as antioxidant responses have been analysed in *Ginkgo* seedlings [11]. Previous work has also shown that male *Ginkgo* plants exhibit stronger vegetative growth at the seedling stage [12]. However, the developmental dynamics of PSII function during leaf growth and natural senescence in *Ginkgo*, and whether PSII functional development exhibits sexual dimorphism under natural conditions, remain largely unreported. In this study, non-destructive OJIP fluorescence induction kinetics and JIP-test analysis were used to systematically characterise PSII development and decline in male and female *Ginkgo* leaves throughout the growing season. This study aims to advance understanding of sex-related differences in PSII functional dynamics during leaf development and senescence in *Ginkgo*, with implications for photosynthesis research and for broader comparative studies of physiological dimorphism in dioecious plants.

## 2. Materials and Methods

### 2.1. Plant Materials and Sampling

**Figure 1:**
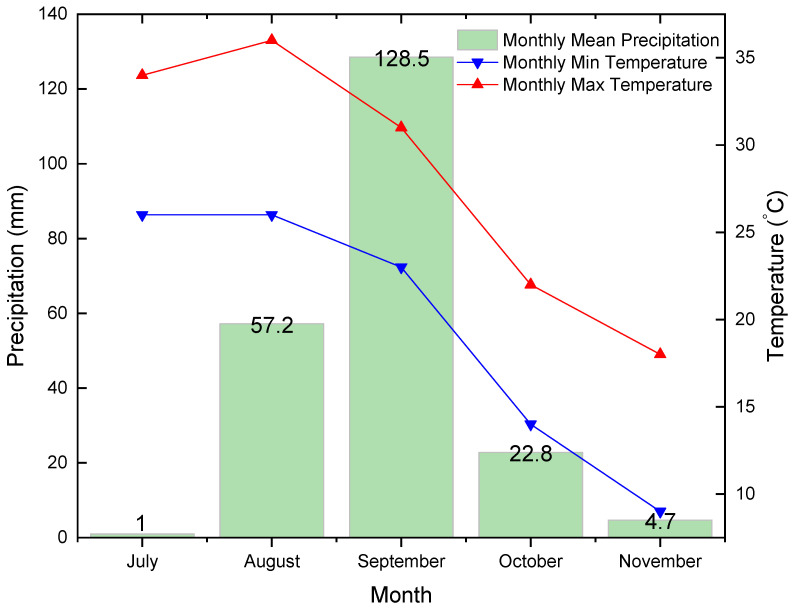
Monthly mean precipitation (bars) and monthly maximum (red line) and minimum (blue line) air temperatures.

This study was conducted using naturally grown male and female *Ginkgo biloba* trees at Nanjing Forestry University. Sampling was conducted from 18 July to 26 November 2024 [day of year (DOY) 188–332] at approximately 15-day intervals. For chlorophyll content determination, healthy and undamaged sun-exposed leaves were newly collected at each sampling date from mature trees (>20 years old) at the same canopy height and from the sun-exposed side of the crown. For chlorophyll fluorescence measurements, the same leaves were monitored across sampling dates as far as possible; when leaf abscission occurred, a nearby leaf at the original position was selected for subsequent measurements. Three biological replicates were used for each sex in both measurements, and mean values were used for subsequent analyses. The climatic conditions recorded during the study period are presented in Figure 1.

### 2.2. Determination of Chlorophyll Content

Chlorophyll content was determined as follows. Fresh leaves were collected, and 0.2 g of leaf tissue was accurately weighed and cut into small pieces. The samples were extracted in the dark with 10 mL of an ethanol: acetone mixture (1:1, *v*/*v*) at room temperature until complete decolorization. The absorbance of the extract was measured at 645 and 663 nm using a UV–Vis spectrophotometer (UV-1800; Shimadzu Corporation, Kyoto, Japan). Total chlorophyll content was calculated according to the equation described by Li et al. [13]:

Total Chl = 20.20 *A*_645_ + 8.02 *A*_663_. (1)

The final chlorophyll content was expressed on a fresh weight basis (mg g^−1^ FW) and used for subsequent statistical analysis.

### 2.3. Measurement of Chlorophyll Fluorescence and JIP-Test Parameters

Chlorophyll fluorescence of intact Ginkgo leaves was measured using a portable continuous-excitation chlorophyll fluorometer (Handy PEA+; Hansatech Instruments Ltd., King’s Lynn, Norfolk, UK). Measurements were conducted at 10:00 a.m. on the same leaves across sampling dates as far as possible, with three biological replicates per sex. When a monitored leaf had abscised, a nearby leaf at the original position was selected for subsequent measurements. Leaves were dark-adapted for 25 min, followed by illumination with a saturating red pulse (3000 μmol photons m^−2^ s^−1^). The instrument automatically recorded high-resolution fluorescence signals from 10 μs to 1 s to obtain the O–J–I–P induction transient. OJIP transient signals were processed using PEA+ software (Version: 1.13) supplied with the instrument (Hansatech Instruments Ltd., King’s Lynn, Norfolk, UK; https://www.hansatech-instruments.com/ (accessed on 12 April 2024)) and Microsoft Excel 2019 (Microsoft Corporation, Redmond, WA, USA), from which fluorescence parameters were derived based on JIP-test analysis. Different kinetics of ΔW_oj_ and ΔW_ok_ were obtained from the differences between double-normalized fluorescence curves in the O–J and O–K phases, respectively. Formulae and definitions for the parameters used are provided in Table 1.

### 2.4. Data Analysis

Chlorophyll fluorescence parameters were calculated using PEA+ software supplied with the Handy PEA+ fluorometer (Hansatech Instruments Ltd., King’s Lynn, Norfolk, UK; https://www.hansatech-instruments.com/). Data processing and visualization were carried out using Microsoft Excel 2019 (Microsoft Corporation, Redmond, WA, USA) and OriginPro 2021 (OriginLab Corporation, Northampton, MA, USA). Statistical analyses were performed using IBM SPSS Statistics 26.0 (IBM Corp., Armonk, NY, USA). Data are presented as mean ± SE. For chlorophyll content, differences among sampling dates within each sex were assessed using one-way ANOVA followed by Tukey’s post hoc test (*p* < 0.05), and sex differences on the same sampling day were evaluated using independent-samples *t* tests. In addition, two-way ANOVA was performed to evaluate the effects of sex, sampling time, and sex × time interaction on chlorophyll content and chlorophyll fluorescence parameters across the full sampling period. Principal component analysis (PCA) was conducted using selected OJIP-derived fluorescence parameters to evaluate multivariate variation and sex-specific patterns during natural senescence.

## 3. Results

### 3.1. Male and Female Differences in Chlorophyll Content of Ginkgo

During the study period, monthly precipitation reached its highest level in September and then declined markedly, while air temperature decreased progressively from August to November (Figure 1).

**Figure 2:**
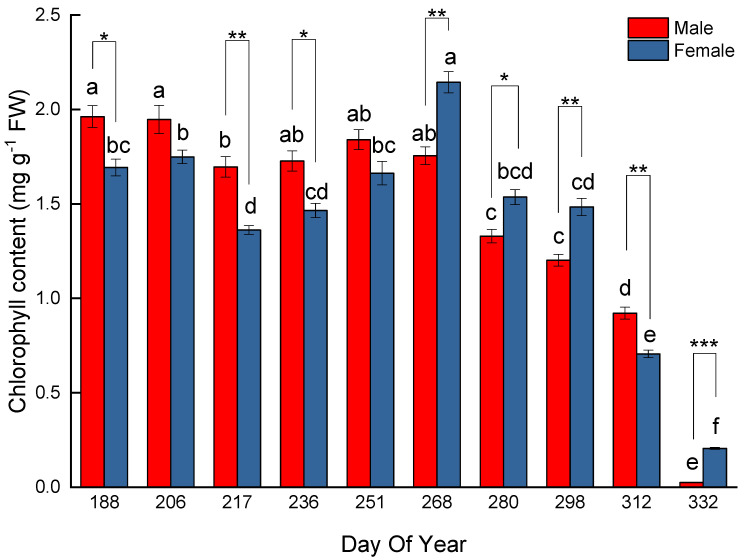
Temporal changes in chlorophyll (Chl) content in male and female samples. Data are presented as mean ± SE (*n* = 3). Bars of different colors represent male and female samples, respectively. For chlorophyll content data, sex differences on the same sampling day were evaluated using independent-samples *t* tests and are indicated by asterisks (* *p* < 0.05, ** *p* < 0.01, *** *p* < 0.001). Different lowercase letters above the bars indicate significant differences among sampling days within the same sex, as determined by one-way ANOVA followed by Tukey’s post hoc test (*p* < 0.05). Bars sharing at least one common letter are not significantly different.

Significant differences in chlorophyll content between male and female leaves were observed throughout leaf development and senescence (Figure 2). Between DOY 188 and 251, chlorophyll content was consistently higher in male leaves than in female leaves, with significant differences observed at most sampling dates (DOY 188, 217, and 236). The chlorophyll content of male plants began to decline from DOY 251, whereas the chlorophyll content of female plants began to decline from DOY 268. A marked decline in chlorophyll content became evident from around DOY 268 in both sexes. However, after DOY 268, chlorophyll content declined more rapidly in male plants than in female plants. By DOY 332, the chlorophyll content of male plants was only 1.37% of that at DOY 268 and 3.46% of that at DOY 312, whereas the chlorophyll content of female plants was 9.55% and 20.13% of the values at DOY 268 and DOY 312, respectively.

### 3.2. Male and Female Differences in OJIP Transients During Natural Senescence in Ginkgo

**Figure 3:**
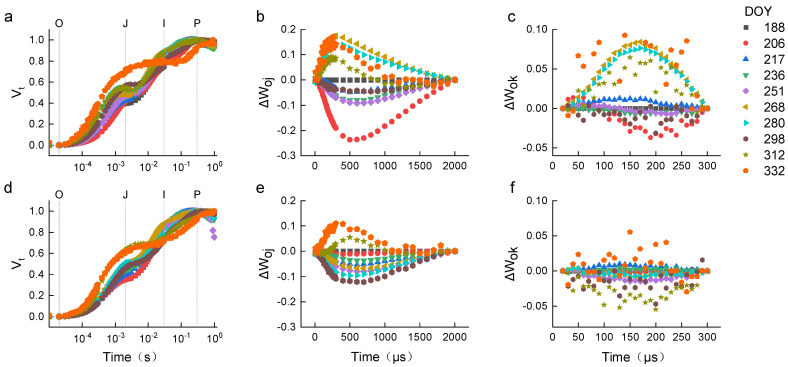
Fast chlorophyll fluorescence transients (OJIP curves) and difference kinetics of *Ginkgo biloba* leaves measured on different days of year (DOY). Panels (**a**–**c**) represent male plants, whereas panels (**d**–**f**) represent female plants. Panels (**a**,**d**) show normalized OJIP fluorescence transients V_t_. Panels (**b**,**e**) show difference kinetics of ΔW_oj_, calculated as the difference between the double-normalized fluorescence curves in the O–J phase, and panels (**c**,**f**) show difference kinetics of ΔW_ok_, calculated as the difference between the double-normalized fluorescence curves in the O–K phase. Different symbols and colors indicate measurements conducted on different DOY, as shown in the legend.

The measured fluorescence intensities were normalized, and the relative variable fluorescence curves (Vt) were plotted separately for female and male plants (Figure 3a,d). Under natural conditions, the fluorescence induction curves of both female and male plants showed the typical OJIP transient. However, differences among measurement dates were still observed at specific phases of the curves. During the OJ phase (0.00002–0.002 s), the separation among the curves measured on different dates was generally more pronounced in male plants than in female plants. In female plants, the J step showed a clear increase on DOY 312 and DOY 332, whereas in male plants, this increase was observed only on DOY 332. In the JI phase, the curves of male plants showed larger differences among measurement dates compared with female plants. The fluorescence curves in male plants were clearly separated in this phase, and some curves displayed an evident elevation. In contrast, the curves of female plants were more closely grouped in the JI phase and showed a relatively consistent rise. After DOY 312, the relative variable fluorescence (Vt) of female plants showed an increase at the J step and a decrease at the I step. In contrast, the curves near the P step were generally similar among different measurement dates, with only small differences in the maximal fluorescence level.

### 3.3. Male and Female Differences in ΔW_oj_ and ΔW_ok_ of Ginkgo

In male plants, ΔW_oj_ exhibited pronounced temporal variation. Before DOY 251, ΔW_oj_ showed negative deviations, whereas after DOY 268 it shifted to positive deviations (except DOY 298) and reached higher peaks than in females on the same dates (Figure 3b,e). This temporal pattern coincided with the decline in chlorophyll content in male plants. Female plants exhibited a smaller ΔW_oj_ amplitude overall. Although slight deviations from zero were detectable, peak values were constrained and returned to near zero more rapidly, and differences among sampling dates were relatively weak. In addition, although female chlorophyll content began to decline at DOY 268, the ΔW_oj_ response remained small until DOY 312.

In male plants, ΔW_ok_ exhibited pronounced upward curvature on DOY 268 and DOY 280, whereas positive deviations were also present on DOY 312 and DOY 332 but were more variable (Figure 3c,f). Female plants did not show obvious sustained upward curvature in ΔW_ok_, but exhibited stronger fluctuations and dispersion than males after DOY 298. Overall, female plants exhibited reduced KI-phase amplitude and mixed positive and negative responses.

### 3.4. Male and Female Differences in Photosynthetic Energy and Electron Fluxes of Ginkgo

**Figure 4:**
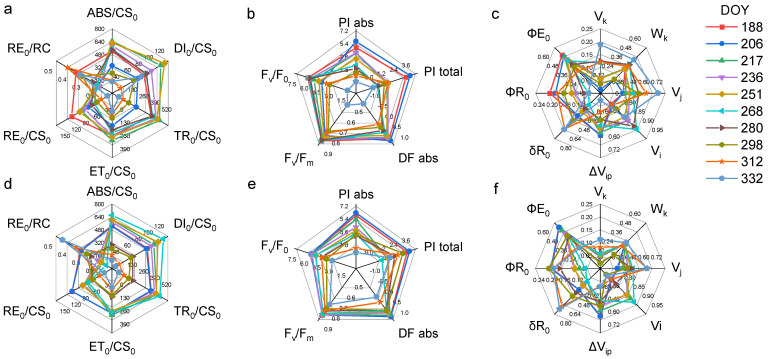
Radar plots of OJIP-derived chlorophyll fluorescence parameters in *Ginkgo biloba* leaves measured on different days of year (DOY). Panels (**a**–**c**) represent male plants, whereas panels (**d**–**f**) represent female plants. Panels (**a**,**d**) show photosynthetic energy and electron fluxes, panels (**b**,**e**) show photosynthetic performance indices, and panels (**c**,**f**) show energy flux and reaction center characteristics. Different symbols and colors indicate measurements conducted on different DOY, as shown in the legend.

During natural senescence, the cross-section-based photosynthetic energy and electron flux parameters of male and female *Ginkgo biloba* leaves changed markedly over time, and the differences between the sexes became more apparent at the later stage of senescence (Figure 4a,d). DOY 268 was used as the dividing point, and the photosynthetic parameters of male and female plants showed a significant stage change. Before DOY 268, the chlorophyll content of both male and female plants remained high, and ABS/CS_0_ values were also relatively high. However, the values of ABS/CS_0_, TR0/CS_0_, and ET_0_/CS_0_ in female plants were higher than those in male plants at this stage. In addition, RE_0_/CS_0_ remained at a relatively high level in female plants, whereas it showed a decreasing trend in male plants.

After DOY 268, the chlorophyll content of both male and female plants decreased, and ABS/CS_0_ also declined. In male plants, TR_0_/CS_0_, ET_0_/CS_0_, and RE_0_/CS_0_ decreased rapidly after DOY 268, whereas DI_0_/CS_0_ remained at a relatively high level. In contrast, although ABS/CS_0_ in female plants also showed a decreasing trend, the declines in TR_0_/CS_0_ and ET_0_/CS_0_ were more gradual, and RE_0_/CS_0_ remained relatively higher at the later stage. From DOY 268 to DOY 332, RE_0_/CS_0_ decreased by 73.11% in male plants and by 67.94% in female plants. In addition, DI_0_/CS_0_ decreased by only 11.92% in male plants over the same period, whereas in female plants it decreased by 76.37% (Table S1,S2). At the later stage of senescence, female plants retained relatively higher RE_0_/CS_0_ values, whereas male plants retained relatively higher DI_0_/CS_0_ values.

### 3.5. Male and Female Differences in Photosynthetic Performance Indices of Ginkgo

During DOY 188–251, DF abs, PI total, PI abs, F_v_/F_m_ and F_v_/F_0_ in both male and female plants remained at relatively high levels (Figure 4b,e), and only small changes were observed with time. Overall, the values of DF abs and PI parameters in female plants were slightly higher than those in male plants at most measurement dates, indicating that female plants had higher overall photosynthetic performance. In addition, the parameters related to the maximum photochemical efficiency of PSII in both male and female plants remained relatively stable during this stage.

After DOY 268, the above fluorescence parameters in both male and female plants showed a decreasing trend, but significant differences between the sexes were observed (Tables S1 and S2). The parameters in male plants showed a rapid decrease after DOY 268. By DOY 312, DF abs had decreased to negative values, and PI total and PI abs reached the lowest levels. At the same time, F_v_/F_m_ and F_v_/F_0_ showed a significant decrease, indicating that the efficiency of energy conversion and the potential activity of PSII were reduced. In contrast, although the parameters in female plants also showed a continuous decrease after DOY 268, the decrease was slower. DF abs decreased to below 0 only at DOY 332, and on the same date the values of PI total and PI abs remained higher than those in male plants, indicating that the overall performance of the photosynthetic apparatus in female plants remained higher at the later stage.

### 3.6. Male and Female Differences in Energy Flux and Reaction Center Characteristics of Ginkgo

During DOY 188–251, the JIP-test parameters in both male and female plants showed small changes (Tables S1 and S2). The values of W_k_, V_j_, V_I_, and V_k_ remained at relatively low levels, whereas ΦR_0_, ΦE_0_, and ΔV_IP_ remained at relatively high levels. During this stage, the values of the parameters in male and female plants were similar, and small differences were observed at some measurement dates, indicating that the structure and function of the photosynthetic apparatus remained relatively stable in both male and female plants during this period.

After DOY 268, the JIP-test parameters in both male and female plants showed significant changes, and significant differences between sexes were also observed in the magnitude and direction of these changes (Table S1, S2). In male plants, after DOY 268, W_k_, V_j_ and V_k_ showed an increase, whereas ΦR_0_, ΦE_0_ and ΔV_IP_ showed a continuous decrease. At the same time, the fluctuations of δR0 and V_I_ became more pronounced. In contrast, although female plants also showed increases in W_k_, V_j_ and V_k_ and decreases in ΦR_0_, ΦE_0_ and ΔV_IP_ after DOY 268, the overall changes were smaller. The values of ΦR_0_ and ΦE_0_ in female plants remained higher than those in male plants during DOY 268–332, and the decrease in ΔV_IP_ was slower. At the same time, δR_0_ and V_i_ did not show a consistent unidirectional increase or decrease, but their temporal variation became more pronounced in male plants. (Figure 4c,f).

These results indicate that, before DOY 268, the photosynthetic apparatus in both male and female plants remained relatively stable. After DOY 268, however, the parameters showed unfavorable changes, and the changes were more pronounced in male plants.

### 3.7. Effects of Sex, Sampling Time, and Their Interaction on Chlorophyll Content and Chlorophyll Fluorescence Parameters

Two-way ANOVA showed that all measured variables varied significantly with sampling time (*p* < 0.001; Table 2). Significant sex effects were detected for several chlorophyll fluorescence parameters, particularly those related to performance indices, early fluorescence transients, and energy fluxes, including DF abs, PI total and PI abs. Significant sex × time interactions were also observed for chlorophyll content and all fluorescence parameters, indicating that the temporal patterns of these traits differed between male and female *Ginkgo biloba* plants during natural senescence. Overall, these results showed that the differences between sexes were reflected more strongly in their seasonal trajectories than in simple overall mean differences.

### 3.8. Principal Component Analysis

**Figure 5:**
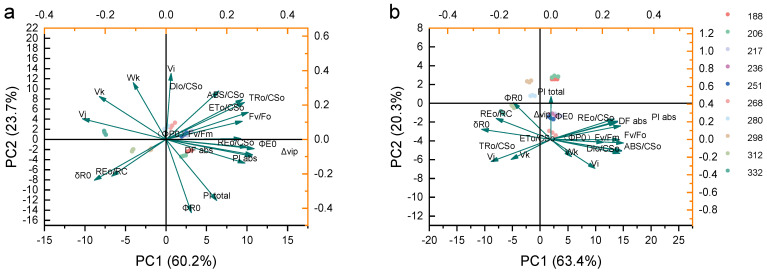
Principal component analysis (PCA) of OJIP-derived chlorophyll fluorescence parameters in *Ginkgo biloba* leaves. (**a**) PCA biplot for male plants. (**b**) PCA biplot for female plants. Dots indicate individual samples measured on different days of year (DOY), and different colors represent different DOY, as shown in the legend. Arrows represent the loading vectors of fluorescence parameters and indicate their contributions to the principal components.

Principal component analysis indicated that the first two principal components adequately explained the variation in OJIP-derived fluorescence parameters in both sexes. In male plants, PC1 and PC2 accounted for 60.2% and 23.7% of the total variance, respectively. In female plants, PC1 and PC2 accounted for 63.4% and 20.3% of the total variance, indicating that the first two principal components explained a comparable proportion of total variance in both sexes (Figure 5).

Principal component analysis (PCA) showed that the first two principal components explained most of the variation in the chlorophyll fluorescence parameters of male and female *Ginkgo biloba* plants. In male plants, positive loadings on PC1 were mainly associated with ΦE_0_ and DF abs, whereas negative loadings were related to V_j_ and δR_0_, showing that PC1 was mainly associated with variation in these parameters. Samples collected at different dates were clearly separated along PC1, indicating temporal variation in the multivariate fluorescence profile during the season. In contrast, in female plants, ET_0_/CS_0_ and TR_0_/CS_0_ contributed most strongly to positive loadings on PC1, while δR_0_ and V_j_ were associated with negative loadings. Female samples tended to cluster more closely along this axis, although differences among sampling dates were still evident.

Across both sexes, PC2 showed strong positive loadings for PI total and ΦR_0_ together with negative loadings for V_I_, V_k_, W_k_, and δR_0_, showing that this component summarized variation among these fluorescence-related parameters. The contrasting loading patterns observed on PC1 between male and female plants showed different multivariate associations among fluorescence parameters in the two sexes.

## 4. Discussion

The integrity of the photosynthetic electron transport chain is essential for maintaining the stability of the photosystems in leaves. During leaf senescence, changes in different steps of the electron transport chain may influence the rate at which overall photosynthetic performance declines. Leaf senescence typically begins with the loss of chlorophyll and the gradual degradation of antenna complexes. These processes reduce the capacity for light absorption and place increasing pressure on downstream electron transport processes [14]. In the present study, the decline in chlorophyll content was observed from DOY 268 in both sexes, with an earlier and more rapid decline in males. A previous transcriptomic study of *Ginkgo* autumn coloration and senescence showed that chlorophyll biosynthesis-related genes declined whereas chlorophyll degradation- and senescence-associated genes increased as leaves progressed toward yellowing [15]. These findings provide molecular context for the chlorophyll decline observed here. However, whether the earlier chlorophyll loss in male plants is associated with differential regulation of chlorophyll biosynthesis- and degradation-related genes remains to be determined. Meanwhile, although ABS/CS0 declined markedly from DOY 268 in both sexes, females maintained higher values, which may indicate a relatively greater stability of the antenna system during senescence. In perennial woody plants, variation in antenna maintenance during senescence can strongly influence the accumulation of photosystem damage [16], which may partly explain the slower functional decline observed in female *Ginkgo*. Seasonal changes in temperature and precipitation may also have contributed to the observed decline in chlorophyll content and PSII-related parameters, because temperature and water availability are known to influence leaf senescence progression and photosynthetic performance under field or stress-related conditions [7,17,18]. Similar consideration of seasonal climate conditions has also been adopted in previous studies of autumn senescence. For example, Holland et al. discussed OJIP fluorescence changes during autumn senescence in relation to seasonal temperature conditions in European oaks [19]. In the present study, the onset of chlorophyll decline in male plants from DOY 251 and in female plants from DOY 268 coincided with the seasonal transition from late summer to autumn, during which air temperature declined markedly after September and precipitation decreased after reaching a peak in September (Figure 1). This temporal correspondence suggests that decreasing temperature and changing water conditions may have promoted the progression of natural senescence. Because both male and female trees were exposed to the same ambient environment, the sex-specific differences observed in chlorophyll decline and OJIP-derived parameters are more likely to reflect different physiological responses under shared seasonal conditions rather than climatic effects alone.

After light absorption, the trapping efficiency of PSII reaction centers largely determines the entry of electrons into the transport chain. Parameters such as F_v_/F_m_, F_v_/F_0_, and TR_0_/CS_0_ are therefore widely used to assess maximal PSII photochemical efficiency and reaction-center activity [20,21]. Previous studies have shown that chlorophyll fluorescence parameters in *Ginkgo biloba* respond sensitively to changes in light environment, indicating that PSII activity and overall photosynthetic efficiency are strongly influenced by environmental conditions [22]. In the present study, these parameters remained high and relatively stable from DOY 188 to 251 in both sexes, suggesting that PSII function was generally maintained during this period. As senescence progressed, however, significant differences between sexes began to emerge. After DOY 268, male plants showed more pronounced decline, whereas females still maintained relatively high values even at DOY 332. Excess light can induce photoinhibition of PSII, usually expressed as a reduction in F_v_/F_m_ [23]. In contrast to acute photo inhibitory damage, the declines in maximal PSII efficiency during natural senescence typically occur gradually [2]. In our study, females maintained higher photochemical efficiency during the late stage of senescence, indicating greater stability of PSII function.

During senescence, the acceptor side of PSII is often considered one of the more sensitive segments of the electron transport chain, and OJIP-derived parameters such as ΔW_oj_ and V_j_ are commonly used as indicators of changes in electron transfer from QA^−^ to QB and the plastoquinone pool [24]. In general, a positive shift in ΔW_oj_ together with an increase in V_j_ reflects enhanced accumulation of QA^−^ and stronger restriction of electron transfer beyond QA^−^. Chlorophyll fluorescence analysis can capture such alterations in PSII behavior, and similar fluorescence responses have also been reported under abiotic stresses such as drought and salinity [25]. In the present study, male plants showed a clear shift in ΔW_oj_ to positive values after DOY 268, accompanied by a marked increase in V_j_, whereas female plants maintained a much smaller ΔW_oj_ response until DOY 312 and showed a weaker increase in V_j_ during most of the senescence period. These changes indicated that acceptor-side restriction in male plants appeared earlier and was more pronounced, while in female plants the restriction was weaker and became evident only at the later stage of senescence. Consistently, female plants maintained relatively higher ET_0_/CS_0_ and ΦE_0_ values, indicating that electron transport flux and quantum yield of electron transport were better preserved in female plants during most of the senescence period. Because acceptor-side restriction may accelerate PSII damage and enhance senescence effects [26], the greater stability of this step in females may contribute to their slower functional decline.

Beyond the acceptor side, donor-side behavior is also relevant to the maintenance of PSII function during senescence. Parameters such as W_k_ and ΔW_ok_ are frequently discussed in relation to donor-side perturbation and possible changes in the oxygen-evolving complex (OEC) [27,28]. In general, an increase in W_k_ together with the appearance of a positive ΔW_ok_ (K-band) reflects impaired electron donation from the OEC to the PSII reaction center, indicating greater OEC-related damage or dissociation [27,28]. In the present study, male plants showed a more evident positive ΔW_ok_ after DOY 268, especially on DOY 268 and DOY 280, whereas female plants exhibited smaller fluctuations without sustained abnormal elevation. At the same time, W_k_ increased more strongly and F_v_/F_m_ declined more markedly in male plants, while female plants maintained comparatively lower W_k_ values and relatively higher F_v_/F_m_ at the later stage of senescence. These patterns suggest that OEC-related damage was less severe in female plants than in male plants during the late stage of senescence. Because donor-side impairment can restrict the supply of electrons entering the photosynthetic electron transport chain and thereby aggravate the downstream decline in electron transport [28], the weaker donor-side disturbance in female plants may contribute to their relatively slower functional decline.

The continuity of photosynthetic electron flow largely depends on the functional status of PSI and the capacity of downstream electron acceptors [29]. Parameters such as δR0 and ΦR_0_ reflect the efficiency and quantum yield of electron transfer from reduced intersystem electron acceptors to the PSI end acceptors, whereas RE_0_/CS_0_ represents the electron flux reducing PSI end acceptors per cross section [30]. In addition, the I–P phase and its relative amplitude (ΔV_IP_) have been associated with PSI-related changes, including variation in PSI acceptor-side capacity and PSI content [30,31]. In the present study, these parameters declined more markedly in male plants from DOY 268 onward, whereas female plants maintained relatively higher δR_0_, ΦR_0_, and RE_0_/CS_0_ values and showed a more gradual decrease in ΔV_IP_. These changes suggest that the transfer of electrons from PSII through the intersystem chain to PSI end acceptors was more strongly restricted in male plants during the late stage of senescence. Because reduced PSI acceptor-side capacity may promote over-reduction in the electron transport chain and aggravate photochemical imbalance [32], the relatively higher values retained in female plants suggest better maintenance of downstream electron transport during senescence. This interpretation is also consistent with the relatively higher TR_0_/CS_0_, ET_0_/CS_0_, and RE_0_/CS_0_ values observed in female plants at the late stage of senescence.

Integrated indices such as PI abs, PI total, and DF abs summarize changes across multiple steps of the electron transport chain. Previous ecological studies have shown that JIP-test-derived parameters can effectively reflect systemic changes in the photosynthetic apparatus and therefore serve as reliable indicators of overall plant vitality and stress responses [33]. In the present study, both sexes showed declines in these indices from DOY 268; however, the decrease was more pronounced in males. In particular, DF abs values in male plants became negative earlier, whereas female plants remained above zero until DOY 332. This further indicates that female plants are able to maintain photosynthetic function more effectively during senescence.

Two-way ANOVA showed significant effects of sampling time and significant sex × time interactions, indicating that male and female *Ginkgo biloba* plants differed in their seasonal trajectories across the sampling period rather than only in overall mean values. PCA provided a descriptive multivariate overview consistent with these differences, with samples from different senescence stages being separated along the principal components and the distinction between sexes becoming more evident in the late stage of senescence. In male plants, the PCA pattern was more strongly associated with chlorophyll content, DF abs, PI abs, PI total, and electron-transport-related parameters, indicating that the decline in PSII performance after DOY 268 appeared earlier and was more pronounced. In contrast, in female plants, ET0/CS0 and TR0/CS0 contributed more strongly to the PCA pattern, indicating that variation in cross-section-based energy trapping and electron transport played a larger role in the seasonal profile of female plants. Together, these results show that natural senescence in *Ginkgo biloba* involves sex-dependent regulation of photosystem deterioration, with male plants showing an earlier and more pronounced decline, whereas female plants maintained photosynthetic function for a longer period.

Sexual dimorphism in dioecious plants is often associated with sex-dependent differences in photosynthetic traits, stress responses, and resource-use strategies, and similar patterns have also been reported in other dioecious species [34,35]. In this context, the present results suggest that the developmental trajectory and senescence-related decline of the photosynthetic apparatus in *Ginkgo biloba* can reflect sexual dimorphism at the physiological level. Compared with male plants, female plants maintained higher chlorophyll content, more stable PSII photochemical efficiency, and a slower decline in electron transport capacity during the late stage of senescence. Therefore, the observed sex-specific differences are not only limited to the timing of senescence onset, but also involve distinct patterns of photosystem functional regulation during seasonal leaf aging.

It should be noted that chlorophyll content and chlorophyll fluorescence measurements followed different sampling structures in the present study, and only three biological replicates were used for each sex at each sampling date. Therefore, the statistical comparisons were intended primarily to describe seasonal trends and sex-specific differences, and the results should be interpreted with caution.

## 5. Conclusions

This study utilised OJIP fluorescence kinetics and JIP-test analysis under natural growth conditions to reveal pronounced sexual dimorphism in the PSII–PSI functional decline pathway during leaf senescence in *Ginkgo biloba*. Although both sexes began senescing around DOY 268, male plants exhibited earlier and stronger limitations on both the acceptor and donor sides, accompanied by a rapid reduction in electron transport to PSI end acceptors and sharper declines in integrated performance indices. In contrast, female plants maintained relatively higher antenna function, cross-section-based energy fluxes, and PSI end-acceptor reduction capacity, resulting in a slower deterioration of overall photosynthetic performance.

Overall, these results indicate significant sex-specific differences in photosystem regulation during senescence in *Ginkgo*. These differences support the existence of sexual dimorphism in the seasonal developmental trajectory and functional decline of the photosynthetic apparatus in this dioecious species. The greater stability of light absorption, electron transport, and downstream electron acceptor capacity in female plants may contribute to their slower decline in photosynthetic performance. These findings provide a physiological basis for understanding sex-specific variation in photosynthetic decline during natural senescence in *Ginkgo*, and also suggest a possible direction for future studies to explore early, non-destructive sex-related screening in *Ginkgo* before reproductive traits become visible. In addition, they may provide a useful reference for comparative studies of sex-specific senescence processes in other dioecious plants.

## Figures and Tables

**Table 1:** Formulae and definitions of selected JIP-test fluorescence parameters used in this study. Subscript “0” indicates that the parameter refers to the onset of illumination, when all reaction centers (RCs) are assumed to be open.

Terms and Formulae	Illustrations
F_0_	Minimum fluorescence yield when all reaction centers are open
F_m_	Maximum fluorescence yield when all reaction centers are closed
F_j_	Fluorescence intensity at the J-step of the fluorescence induction curve
F_k_	Fluorescence intensity at the K-step of the fluorescence induction curve
F_i_	Fluorescence intensity at the I-step of the fluorescence induction curve
V_j_ = (F_j_ − F_0_)/(F_m_ − F_0_)	Relative variable fluorescence at phase J of the fluorescence induction curve
V_i_ = (F_i_ − F_0_)/(F_m_ − F_0_)	Relative variable fluorescence at phase I of the fluorescence induction curve
V_k_ = (F_k_ − F_0_)/(F_m_ − F_0_)	Relative variable fluorescence at phase K of the fluorescence induction curve
W_k_ = (F_k_ − F_0_)/(F_j_ − F_0_)	Indicator of oxygen-evolving complex (OEC) perturbation
ΔV_ip_ = (F_m_ − F_i_)/(F_m_ − F_0_)	Relative amplitude of the I-P phase
RE_0_/RC = M_0_ (1/V_j_)(1 − V_i_)	Electron transport from Q–A to the PSI electron acceptors
F_v_/F_0_ = (F_m_ − F_0_)/F_0_	Ratio of variable to minimal fluorescence, reflecting the potential activity of photosystem II reaction centers
ΦP_0_ = TR_0_/ABS = F_v_/F_m_ = [1 − (F_0_/F_m_)]	Maximum quantum yield of primary photochemistry
ΦE_0_ = ET_0_/ABS = [1 − (F_0_/F_m_)] × (1 − V_j_)	Quantum yield of electron transport
δR_0_ = (1 − V_i_)/(1 − V_j_)	Efficiency with which an electron can move from the reduced intersystem electron acceptors to the PSI end electron acceptors
ΦR_0_ = ΦP_0_ (1 − V_j_) δR_0_	Quantum yield of reduction in end electron acceptors of PSI
ABS/CS_0_ = F_0_	Absorption flux of photons per cross section (at t = 0)
TR_0_/CS_0_ = ΦP_0_ × (ABS/CS_0_)	Trapped energy flux per cross section (at t = 0)
ET_0_/CS_0_ = ΦP_0_ × ψ_0_ × (ABS/CS_0_)	Electron transport flux per cross section (at t = 0)
DI_0_/CS_0_ = (ABS/CS_0_) − (TR_0_/CS_0_)	Dissipation energy flux per cross section (at t = 0)
RE_0_/CS_0_ = (ABS/CS_0_) × ΦR_0_	Electron flux reducing end electron acceptors at the PSI acceptor side per cross section
PI abs = (RC/ABS) × [ΦP_0_/(1 -ΦP_0_)] × [ψ_0_/(1 − ψ_0_)]	Performance index on absorption basis
D.F. = log(PI abs)	Driving force on absorption basis DF abs = log(PI abs)
PI total = PI abs × [δR_0_/(1 − δR_0_)]	Total performance index on absorption basis

**Table 2:** Summary of two-way analysis of variance for chlorophyll content and chlorophyll fluorescence parameters in male and female *Ginkgo biloba* plants across different sampling times, including (1) sex effects, (2) time effects, and (3) sex × time interaction Abbreviations: least squares mean, LSM; standard error, SE; degrees of freedom, df; F statistic, F; probability value, P. Effects with *p* < 0.10 are denoted by an asterisk (*).

Variable	Male LSM ± SE	Female LSM ± SE	Sex df	Sex F	Sex P	Time df	Time F	Time P	Sex × Time df	Sex × Time F	Sex × Time P	Significant Effects (*p* < 0.10)
Chlorophyll Content	1.441 ± 0.0421	1.401 ± 0.0368	1, 4	0.505	0.517	9, 36	1395.460	<0.001 *	9, 36	80.566	<0.001 *	Time, Sex × Time
DF abs	0.2990 ± 0.00850	0.4992 ± 0.0140	1, 4	149.593	<0.001 *	9, 36	1941.939	<0.001 *	9, 36	193.756	<0.001 *	Sex, Time, Sex × Time
PI total	1.617 ± 0.0376	2.280 ± 0.0674	1, 4	73.938	0.001 *	9, 36	2111.364	<0.001 *	9, 36	323.684	<0.001 *	Sex, Time, Sex × Time
PI abs	2.892 ± 0.0877	3.711 ± 0.1046	1, 4	35.998	0.004 *	9, 36	1675.318	<0.001 *	9, 36	36.200	<0.001 *	Sex, Time, Sex × Time
W_k_	0.3225 ± 0.00913	0.2923 ± 0.00763	1, 4	6.418	0.064 *	9, 36	1004.648	<0.001 *	9, 36	591.426	<0.001 *	Sex, Time, Sex × Time
V_j_	0.5171 ± 0.0153	0.4780 ± 0.0125	1, 4	3.943	0.118	9, 36	761.311	<0.001 *	9, 36	37.794	<0.001 *	Time, Sex × Time
V_I_	0.8220 ± 0.0246	0.7981 ± 0.0214	1, 4	0.536	0.505	9, 36	85.988	<0.001 *	9, 36	16.195	<0.001 *	Time, Sex × Time
δR_0_	0.3895 ± 0.0103	0.4092 ± 0.0115	1, 4	1.613	0.273	9, 36	1967.659	<0.001 *	9, 36	76.103	<0.001 *	Time, Sex × Time
ΔV_IP_	0.4828 ± 0.0132	0.5235 ± 0.0146	1, 4	4.289	0.107	9, 36	849.857	<0.001 *	9, 36	34.776	<0.001 *	Time, Sex × Time
V_k_	0.0833 ± 0.00240	0.0553 ± 0.00160	1, 4	94.302	<0.001 *	9, 36	1843.130	<0.001 *	9, 36	556.870	<0.001 *	Sex, Time, Sex × Time
(ΦP_0_) F_v_/F_m_	0.7909 ± 0.0214	0.8164 ± 0.0213	1, 4	0.710	0.447	9, 36	136.749	<0.001 *	9, 36	39.507	<0.001 *	Time, Sex × Time
F_v_/F_0_	4.451 ± 0.0964	4.635 ± 0.1025	1, 4	1.721	0.260	9, 36	1233.760	<0.001 *	9, 36	233.969	<0.001 *	Time, Sex × Time
ΦR_0_	0.1413 ± 0.00314	0.1647 ± 0.00348	1, 4	24.905	0.008 *	9, 36	1889.012	<0.001 *	9, 36	509.470	<0.001 *	Sex, Time, Sex × Time
ΦE_0_	0.3903 ± 0.00719	0.4298 ± 0.00814	1, 4	13.213	0.022 *	9, 36	3339.911	<0.001 *	9, 36	180.096	<0.001 *	Sex, Time, Sex × Time
RE_0_/RC	0.2675 ± 0.00510	0.2684 ± 0.00554	1, 4	0.013	0.915	9, 36	2704.746	<0.001 *	9, 36	476.116	<0.001 *	Time, Sex × Time
ABS/CS_0_	436.2 ± 8.751	433.7 ± 8.837	1, 4	0.039	0.853	9, 36	1800.377	<0.001 *	9, 36	144.229	<0.001 *	Time, Sex × Time
DI_0_/CS_0_	78.10 ± 1.117	73.28 ± 1.165	1, 4	8.896	0.041 *	9, 36	3996.417	<0.001 *	9, 36	769.130	<0.001 *	Sex, Time, Sex × Time
TR_0_/CS_0_	358.8 ± 5.957	360.0 ± 4.924	1, 4	0.024	0.885	9, 36	6922.757	<0.001 *	9, 36	569.586	<0.001 *	Time, Sex × Time
ET_0_/CS_0_	185.1 ± 2.476	200.0 ± 3.097	1, 4	14.079	0.020 *	9, 36	4039.261	<0.001 *	9, 36	248.408	<0.001 *	Sex, Time, Sex × Time
RE_0_/CS_0_	60.42 ± 0.9299	65.50 ± 0.9015	1, 4	15.402	0.017 *	9, 36	9255.877	<0.001 *	9, 36	722.560	<0.001 *	Sex, Time, Sex × Time

## Data Availability

The data presented in this study are available in the article.

## Supplementary Materials

The following supporting information can be downloaded at: https://www.mdpi.com/article/10.3390/life16071060/s1, Table S1, chlorophyll fluorescence parameters of male *Ginkgo biloba* plants measured on different days of year (DOY), and Table S2, the corresponding parameters of female plants.

## Abbreviation

The following abbreviations are used in this manuscript:

DOY	Day Of Year
