# Supplementary material for: Sex-Specific Seasonal Trajectories of Photosystem II Function During Natural Senescence in Ginkgo biloba Revealed by OJIP Fluorescence Analysis
Source: Life (Basel). 2026 Jun 25;16(7):1060. doi: 10.3390/life16071060 (PMC13412698; doi:10.3390/life16071060)
Supplement: Supplementary file 1 [file life-16-01060-s001.zip › life-4251063-supplementary.pdf]

Table S1. Chlorophyll fluorescence parameters of male *Ginkgo biloba* plants measured on different days of year (DOY) during natural senescence. Data are means ± SE. Different lowercase letters within a row indicate significant differences among sampling dates ($P < 0.05$).

DOY	188	206	217	236	251	268	280	298	312	332
DF abs	0.696±0.022ab	0.764±0.053a	0.611±0.027b	0.623±0.031b	0.519±0.026c	0.377±0.021d	0.286±0.015e	0.132±0.005f	-0.023±0.002g	-0.996±0.050h
PI total	3.376±0.169b	3.740±0.115a	1.485±0.054cd	1.666±0.059c	1.578±0.079cd	0.607±0.027e	0.710±0.035e	1.255±0.045d	1.576±0.064cd	0.175±0.009f
PI abs	4.913±0.319b	5.744±0.287a	4.081±0.183c	4.143±0.219c	3.314±0.166d	2.393±0.120e	1.925±0.096ef	1.354±0.075fg	0.950±0.052g	0.101±0.005h
（ΦP_0）Fv/Fm	0.322±0.016c	0.181±0.009d	0.336±0.017bc	0.321±0.016c	0.324±0.013c	0.381±0.020b	0.378±0.019b	0.183±0.012d	0.359±0.016bc	0.439±0.022a
F_v/F_0	0.409±0.020f	0.407±0.020f	0.436±0.022ef	0.479±0.029cdef	0.475±0.024def	0.501±0.017cde	0.556±0.028bc	0.545±0.031bcd	0.620±0.038b	0.743±0.041a
W_k	0.753±0.023b	0.768±0.039b	0.839±0.056ab	0.854±0.043ab	0.822±0.054ab	0.907±0.059a	0.877±0.044ab	0.794±0.028ab	0.775±0.043b	0.831±0.042ab
V_j	0.410±0.020c	0.396±0.020c	0.273±0.009d	0.284±0.014d	0.324±0.013d	0.204±0.012e	0.269±0.013d	0.474±0.015b	0.626±0.031a	0.634±0.032a
V_i	0.591±0.030a	0.593±0.030a	0.562±0.017ab	0.525±0.029abc	0.525±0.026abc	0.493±0.018bc	0.447±0.027cd	0.449±0.023cd	0.377±0.017d	0.266±0.013e
δR_0	0.049±0.002c	0.046±0.002e	0.050±0.003c	0.058±0.003c	0.053±0.003c	0.058±0.003b	0.024±0.001b	0.021±0.001d	0.082±0.004b	0.112±0.006a
ΔV_{IP}	0.848±0.026a	0.811±0.028a	0.836±0.050a	0.853±0.047a	0.814±0.045a	0.855±0.047a	0.835±0.040a	0.759±0.018a	0.782±0.055a	0.516±0.029b
V_k	5.826±0.205a	4.145±0.157d	5.059±0.290bc	5.482±0.146ab	4.601±0.204cd	5.778±0.116a	5.740±0.282a	3.249±0.113e	3.561±0.145e	1.066±0.046f
ΦR_0	0.207±0.005a	0.191±0.007ab	0.127±0.004d	0.126±0.008d	0.142±0.006d	0.087±0.001e	0.101±0.007e	0.162±0.006c	0.182±0.009b	0.087±0.002e
ΦE_0	0.503±0.013a	0.470±0.017ab	0.476±0.012ab	0.440±0.014bc	0.439±0.013bc	0.423±0.015cd	0.381±0.014de	0.342±0.015e	0.295±0.009f	0.135±0.005g
RE_0/RC	0.360±0.009b	0.201±0.006e	0.211±0.006de	0.190±0.007e	0.207±0.008e	0.214±0.004de	0.239±0.012d	0.308±0.011c	0.419±0.015a	0.327±0.012c
ABS/CS_0	519.513±15.978d	341.26±8.531e	647±29.649a	588±11.76bc	625±27.243ab	555.9±27.25cd	535±16.05cd	244.693±10.023f	161.65±5.111g	144.053±6.078g
DI_0/CS_0	76.150±1.923cd	65.487±1.368ef	107.254±2.124a	89.344±2.264b	113.470±2.846a	81.479±2.024c	81.486±2.037c	59.165±2.071f	35.373±1.040g	71.764±1.880de
TR_0/CS_0	446.595±13.3	278.759±8.19	540.808±14.3	496.396±17.4	518.732±15.6	464.626±12.2	452.540±11.4	189.032±5.67	125.562±4.97	75.197±1.560

	98d	9e	08a	91bc	39ab	55cd	27d	1f	0g	h
ET_0/CS_0	262.958±4.030b	161.498±4.950e	307.803±4.687a	263.364±7.577b	269.606±6.762b	230.528±8.150c	204.373±4.198d	84.366±1.293f	46.053±1.886g	20.352±0.719h
RE_0/CS_0	107.385±2.250a	65.653±2.253d	82.942±2.074b	74.589±1.548c	88.014±2.186b	48.306±1.708f	54.317±1.958e	40.691±0.841g	29.324±1.217h	12.991±0.444i

Table S2. Chlorophyll fluorescence parameters of female Ginkgo biloba plants measured on different days of year (DOY) during natural senescence. Data are means ± SE. Different lowercase letters within a row indicate significant differences among sampling dates ($P < 0.05$).

DOY	188	206	217	236	251	268	280	298	312	332
DF abs	0.771±0.039a	0.780±0.042a	0.731±0.037a	0.621±0.023b	0.584±0.021bc	0.527±0.029cd	0.485±0.024d	0.521±0.033cd	0.113±0.006e	-0.141±0.007f
PI total	3.481±0.174a	3.720±0.187a	1.559±0.057d	1.693±0.076d	1.651±0.106d	1.029±0.037e	2.281±0.125c	3.050±0.154b	2.553±0.169c	1.783±0.089d
PI abs	5.900±0.295ab	6.183±0.410a	5.313±0.224b	4.240±0.212c	3.751±0.137cd	3.400±0.121de	3.067±0.168e	3.230±0.163de	1.301±0.058f	0.722±0.036f
（ΦP_0） Fv/Fm	0.848±0.030a	0.842±0.047a	0.832±0.038a	0.846±0.038a	0.831±0.029a	0.842±0.043a	0.801±0.046a	0.774±0.031a	0.810±0.041a	0.739±0.037a
F_v/F_0	5.411±0.166a	5.433±0.157a	5.364±0.276a	5.394±0.189a	5.006±0.233a	4.933±0.232a	4.181±0.105b	3.583±0.164c	4.115±0.158bc	2.932±0.171d
W_k	0.326±0.016ab	0.321±0.167ab	0.332±0.008ab	0.325±0.012ab	0.315±0.017ab	0.318±0.016ab	0.174±0.008c	0.181±0.009c	0.291±0.013b	0.340±0.017a
V_j	0.363±0.018d	0.357±0.018d	0.406±0.016cd	0.452±0.023bc	0.482±0.024bc	0.508±0.016b	0.496±0.027b	0.451±0.018bc	0.638±0.032a	0.627±0.029a
V_i	0.764±0.038ab	0.760±0.038ab	0.865±0.043ab	0.0841±0.039ab	0.839±0.034ab	0.881±0.049a	0.790±0.027ab	0.743±0.040b	0.759±0.038ab	0.739±0.037b
δR_0	0.370±0.017c	0.373±0.019c	0.230±0.012e	0.285±0.014de	0.300±0.015d	0.234±0.012e	0.427±0.021c	0.495±0.032b	0.672±0.024a	0.707±0.037a
ΔV_{IP}	0.637±0.032a	0.639±0.039a	0.592±0.030ab	0.550±0.030bc	0.521±0.021bc	0.504±0.026c	0.511±0.026c	0.551±0.028bc	0.364±0.006d	0.366±0.018d
V_k	0.059±0.003c	0.016±0.001c	0.059±0.003c	0.057±0.003c	0.057±0.003c	0.118±0.006c	0.124±0.006d	0.036±0.002d	0.119±0.006b	0.188±0.009a
ΦR_0	0.201±0.006a	0.202±0.007a	0.116±0.005c	0.130±0.007c	0.130±0.004c	0.096±0.004d	0.176±0.005b	0.208±0.007a	0.197±0.008a	0.192±0.008ab
ΦE_0	0.540±0.014a	0.542±0.019a	0.508±0.018a	0.463±0.019b	0.433±0.011bc	0.409±0.015c	0.415±0.010c	0.422±0.015bc	0.294±0.007d	0.272±0.015d
RE_0/RC	0.323±0.011c	0.321±0.013c	0.167±0.004gh	0.202±0.007f	0.178±0.008fg	0.139±0.004h	0.253±0.010e	0.286±0.013d	0.363±0.013b	0.451±0.014a
ABS/CS_0	532±15.96c	531.26±15.78c	610.04±26.328ab	573.083±14.471bc	611.05±33.685ab	661.197±27.436a	296.94±13.473d	250.293±9.351d	170±6.129e	101.573±5.235f
DI_0/CS_0	82.789±2.077d	81.271±2.052d	94.004±2.382c	89.960±3.961c	102.468±2.570b	112.070±3.412a	57.093±1.185e	53.715±1.647e	32.988±0.684f	26.486±0.669f
TR_0/CS_0	452.482±9.357c	448.901±11.759c	514.459±10.187b	494.509±14.544b	499.520±12.655b	550.969±8.331a	233.145±8.976d	193.749±5.899e	135.293±3.451f	77.167±1.948g

ET_0/CS_0	286.318±5.726ab	286.819±8.733ab	303.777±6.282a	268.197±9.574b	272.545±14.233b	274.708±4.168b	121.969±3.895c	105.691±4.415c	50.533±1.598 d	29.120±0.996 e
RE_0/CS_0	105.182±2.811a	106.147±1.627a	69.858±1.435 c	76.608±1.903 b	77.897±1.967 b	63.392±1.793 d	50.816±1.301 e	51.666±1.296 e	32.521±1.337 f	20.321±0.508 g
